# Intake of non-nutritive sweeteners is associated with an unhealthy lifestyle: a cross-sectional study in subjects with morbid obesity

**DOI:** 10.1186/s40608-017-0177-x

**Published:** 2017-12-27

**Authors:** Robert Winther, Martin Aasbrenn, Per G. Farup

**Affiliations:** 1 Department of Research, Innlandet Hospital Trust, PB 104, N-2381 Brumunddal, Norway; 2 Faculty of Medicine, University of Aalborg, DK-9100 Aalborg, Denmark; 3 Department of Surgery, Innlandet Hospital Trust, N-2819 Gjøvik, Norway; 4 Unit for Applied Clinical Research, Department of Clinical and Molecular Medicine, Faculty of Medicine and Health Sciences, Norwegian University of Science and Technology, N-7491 Trondheim, Norway

**Keywords:** Diet, General health, Life style, Non-nutritive sweeteners, Obesity

## Abstract

**Background:**

Subjects with morbid obesity commonly use Non-Nutritive Sweeteners (NNS), but the health-related effects of NNS have been questioned. The objectives of this study were to explore the associations between theuse of NNS and the health and lifestyle in subjects with morbid obesity.

**Methods:**

This cross-sectional study included subjects with morbid obesity (BMI ≥ 40 kg/m^2^ or ≥35 kg/m^2^ with obesity-related comorbidity). Information about demographics, physical and mental health, and dietary habits was collected, and a blood screen was taken. One unit of NNS was defined as 100 ml beverages with NNS or 2 tablets/units of NNS for coffee or tea. The associations between the intake of NNS and the health-related variables were analyzed with ordinal regression analyses adjusted for age, gender and BMI.

**Results:**

One hundred subjects (women/men 83/17; mean age 44.3 years (SD 8.5)) were included. Median intake of NNS was 3.3 units (range 0 – 43). Intake of NNS was not associated with BMI (*p* = 0.64). The intake of NNS was associated with reduced heavy physical activity (*p* = 0.011), fatigue (*p* < 0.001), diarrhea (*p* = 0.009) and reduced well-being (*p* = 0.046); with increased intake of total energy (*p* = 0.003), fat (*p* = 0.013), carbohydrates (*p* = 0.002), sugar (*p* = 0.003) and salt (*p* = 0.001); and with reduced intake of the vitamins A (*p* = 0.001), C (*p* = 0.002) and D (*p* = 0.016).

**Conclusions:**

The use of NNS-containing beverages was associated with an unhealthy lifestyle, reduced physical and mental health and unfavourable dietary habits with increased energy intake including sugar, and reduced intake of some vitamins.

## Background

In adults, the global prevalence rates of overweight and obesity, defined as Body Mass Index (BMI) above 25 and 30 kg/m^2^, were in 2014 39% and 13% respectively [1]. The prevalence rates have more than doubled since 1980 and the disorders have been mentioned as one of the largest public health concerns worldwide because of the increased risk of serious non-communicable diseases such as cancer, cardiovascular diseases, and diabetes [1–3]. In Norway, 1 in 4 middle-aged men and 1 in 5 women have a BMI above 30 kg/m^2^ [4].

The “obesity epidemic” (the rapidly increasing prevalence) is caused by environmental and societal changes with increased intake of energy-dense food and increased physical inactivity [1]. Interventions at the societal level should facilitate regular physical activity and make healthier dietary choices available [1]. At the individual level, it is recommended to limit the energy intake from fat and sugar, to increase the intake of fruits, vegetables, legumes, whole grains and nuts, and to increase the regular physical activity [1].

To maintain the pleasure of the sweet taste and at the same time reduce the energy intake, subjects with obesity commonly replace sugar by non-nutritive sweeteners (NNS). The reasoning is logical and the producers of NNS have promoted the use and raised the global market to $ 5.5 billion in 2014 [5]. The effect of NNS on weight prevention and reduction is controversial, and serious safety concerns have been raised [6–11]. The controversies are in part related to the study design. Observational studies indicate weight gain and interventional studies the opposite [12]. Both designs are prone to bias. Bias is also introduced by the industry; the relative risk to have favourable results in industry-sponsored reviews was 17.25 (95%CI 2.34 to 127.29) times that of industry independent ones [2]. Most studies have focused on the effect on body weight, whereas associations with lifestyle and general health have been less studied.

The aims of this study in subjects with morbid obesity were to assess associations between the use of NNS and demographics, lifestyle, physical and mental health, dietary habits, comorbidity and a blood screen.

## Methods

### Study design

This cross-sectional study was performed at the unit for morbid obesity at Innlandet Hospital Trust, Gjøvik, Norway. Consecutive subjects were included from December 2012 through September 2014. A medical history was taken, a physical examination was performed, and a blood sample was collected for further analyses. The patients filled in paper-based questionnaires. A trained study nurse was responsible for the care of the patients and the practical work.

### Subjects

Consecutive subjects aged 18 – 65 years old with a BMI ≥ 40 kg/m^2^ or ≥35 kg/m^2^ with obesity-related complications referred for evaluation of bariatric surgery or conservative treatment were included in a comprehensive study. Subjects with serious somatic and psychiatric disorders judged as unrelated to obesity and subjects with previous major surgery including bariatric surgery were excluded. Only subjects with satisfactorily filled in food frequency questionnaires (FFQ) were included in this study.

### Variables

Demographics: Gender; age (years); body weight (kg), height (meter), body mass index (BMI, kg/m^2^); cohabitant (yes/no); working (no / part-time / full-time); smoking (never / previously / daily); and overall physical activity (score 0 – 8) and heavy physical activity (hours per week: no / <1 / 1-2 / >2).

Diseases, disorders and well-being: Perceived state of health (poor / not quite good / good / very good); present or previous somatic disorders including hypertension, diabetes, and fibromyalgia (yes / no); muscle-skeletal pain score (score 0-12); WHO-5 well-being index (score 0-100; score ≤ 28 = likely depression; score ≤ 50 = low mood); Hopkins Symptom Checklist −10 (HSCL-10) for measurement of mental distress (score 1-4; mental distress ≥1,85); Fatigue severity scale (FSS; score 9-63, score ≥ 36 = fatigue) [13–15]. The functional gastrointestinal disorders Irritable bowel Syndrome (IBS), functional constipation, functional diarrhea, and functional bloating were diagnosed with a validated Norwegian translation of the Rome III criteria; and the degree of gastrointestinal complaints with Gastrointestinal Symptom Rating Scale – IBS (GSRS-IBS) with subscales for GSRS-diarrhea, −constipation and -bloating (scores 1-7) [16, 17].

The dietary intake of nutrients, energy, and NNS was assessed with an FFQ prepared and validated by the Department of Nutrition at the University of Oslo, Norway who also analyzed the FFQs with their in-house calculation program (KBS, version 7.3, food database AE-14) based on the official Norwegian food composition table from 2016 (http://www.matvaretabellen.no). The frequency was reported as less than once/week; 1-2 times/week; 3-4 times/week; 5-6 times/week; once daily; 2 times/day; 3 times/day; ≥ 4 times/day. The portion size was reported in liter (1/5, 1/3: 1/2, 1) and/or glasses and the amounts converted into gram/day. As the FFQ did not capture the type or amount of NNS used in beverages or NNS tablets, the calculation of the NNS intake was performed pragmatically. One unit of NNS was defined as 100 ml NNS-containing beverage (divided into carbonated and non-carbonated beverage). This was considered as the amount of NNS that would equal the sweetening of regular sugar containing beverages with 10% of sugar (10 g/100 ml). One tablet of NNS was approximately equal to 1 teaspoon of sugar (5 g). Thus, 2 NNS tablets/units for use in tea or coffee were judged as equally amount of 100 ml NNS in beverages. 100 ml was chosen as the unit because the subjects reported the intake in liter and/or glasses and the unit is easy to understand. Intakes of NNS from other sources than beverages and tablets used in beverages were not included in the FFQ. Sugar alcohols and naturally-derived sweeteners not defined as NNS were not included. A range of hematological and biochemical blood tests including vitamins and minerals were analyzed.

### Statistics

The results have been reported as mean (SD), median (range), and number (proportion in percentage). Because the intake of NNS varied markedly and was clustered in groups, the intake was ordered in groups with roughly uniform intake and analyzed with ordinal regression analyses. Associations between NNS and the subjects’ characteristics and blood tests were analyzed with ordinal logistic regression analyses adjusted for age, gender and BMI and reported as B- and *p*-values. The associations between NNS and dietary intake were not linear and were analyzed with Spearman’s correlation test reported as rho, and the p-values were calculated with ordinal logistic fractional polynomial regression adjusted for gender, age and BMI. The analyses were performed with IBM SPSS Statistics for Windows, Version 24.0. Armonk, NY: IBM Corp, and the fractional polynomial regression analyses with STATA v14, StatCorp LLC, Texas, USA. *P*-values <0.05 were judged as statistically significant.

### Ethics

The study was approved by the Norwegian Regional Committees for Medical and Health Research Ethics, PB 1130, Blindern, 0318 Oslo, Norway (reference number 2012/966) and performed in accordance with the Declaration of Helsinki. Written informed consent to participate was given by all participants before inclusion.

## Results

**Fig. 1:**
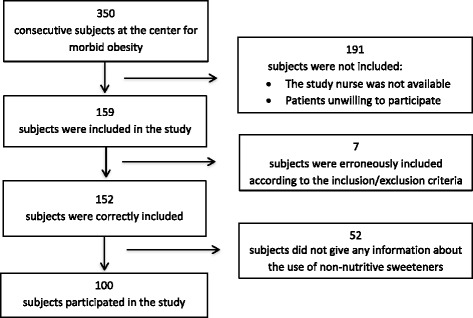
A flow chart of the subjects in the study

**Fig. 2:**
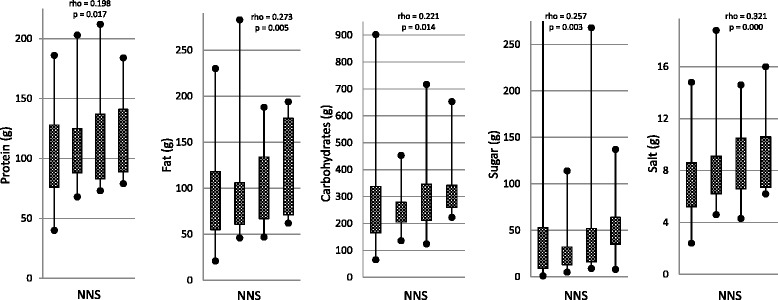
Associations between the intake of NNS in carbonated beverages and intake of nutrients and salt. NNS: Non-Nutritive Sweeteners. The box-and-whisper plots indicate no (0 unit) /low (0.1 – 2.0 units) / medium (2.1 – 9.0 units) / high (9.0 – 40.0 units) intake of non-nutritive sweeteners in carbonated beverages. One unit = 100 ml NNS-beverage/day. The correlations have been calculated with Spearmans’ rho, and the *p*-values with ordinal logistic fractional polynomial regression.

Out of 350 consecutive subjects visiting the obesity unit, 100 (83 women and 17 men with a mean age of 44.3 years (SD 8.5)) were included in the study. The reasons for the exclusion of 250 subjects are given in Fig. 1. Table 1 gives the participants’ characteristics in detail and the results of the blood tests. Table 2 gives the daily dietary intake of energy, energy-yielding nutrients, NNS, vitamins, and salt. The total intake of NNS varied from zero to 43 units per day. High intake of NNS was associated with diabetes, reduced physical activity, fatigue, reduced well-being, and diarrhea (Table 3). Table 4 gives all the associations between intake of NNS and the dietary intake of energy, energy-yielding nutrients, vitamins, and salt. Intake of NNS was associated with increased intake of energy and salt, and reduced intake of vitamins. The positive associations between the intake of NNS and energy and salt were most pronounced for the use of NNS in carbonated beverages and are presented in Fig. 2.

**Table 1:** The characteristics of the participants in the study

Participants’ characteristics (if less than 100, the number is given in brackets)	Mean Median Number	SD Range Proportion (%)
Gender (female/male)	83 / 17	83% / 17%
Age (years)	44.3	8.5
Body weight (kg)	121.8	16.2
BMI (kg/m2)	41.9	3.5
Living with someone (99)	84	85%
Working (no / part time / full-time) (98)	23 / 32 / 43	23%/33%/44%
Smoking (never/previously/daily)	43 /44 / 13	43%/44%/13%
Total physical activity (score 0-8)	4.6	2.2
Heavy physical activity (hrs. Per week: no / <1 / 1-2 / >2)	28/29/32/11	28%/29%/32%/11%
State of health (98) (Poor/Not quite good/ Good/ Very good)	10/54/30/4	10%/55%/31%/4%
Fibromyalgia	19	19%
Muscle-skeletal pain score (range 0-12)	4.0	0 – 12
Hypertension (96)	57	59%
Diabetes	20	20%
HSCL10 > 1.85 (mental distress)	27	27%
WHO-5 (low mood) (cut-off <50)	30	30%
Fatigue (cut-of >36) (99)	48	48%
Irritable bowel syndrome (97)	27	28%
Functional bloating (96)	14	15%
Functional diarrhea (97)	2	2%
GSRS-diarrhea (score 1 - 7) (80)	1.5	1.0 – 4.8
GSRS-bloating (score 1 - 7) (80)	2.3	1.0 – 6.0
Blood tests		
Haemoglobin (F: 11-15; M: 13-17 g/dl) (98)	14.4	1.1
Serum iron (9-34 μmol/L (98)	15.0	5.5
Transferrin saturation (0.10-0.57) (97)	0.23	0.09
Ferritin (10-380 μg/dL) (98)	96	7 - 584
CRP (<5 mg/L) (98)	5	0 - 28
s-Glucose (4.2-6.3 mmol/L) (98)	5.7	4.0 – 23.2
HbA1C (4.3-5.6%) (98)	5.4	4.6 – 11.5
C-peptide (0.3-2.4 nmol/L) (98)	1.47	0.53 – 4.31
Cholesterol (3-7 mmol/L) (98)	5.0	1.0
HDL (F: 1.0-2.7; M: 0.8-2.1 mmol/L) (98)	1.2	0.3
LDL (1-5 mmol/L) (98)	3.3	0.9
Vitamin A (1.2-3.4 μmol/L) (91)	2.0	0.4
Vitamin B1 (122-223 nmol/l) (97)	158	27
Vitamin B6 (27-273 nmol/l) (96)	23	6 - 209
Vitamin B12 (141-700 pmol/L) (98)	338	173 - 1401
Vitamin D (45-161 nmol/L) (98)	58	23
Folic acid (9-36 nmol/l)	17	7 – 46

*HSCL10* Hopkins Symptom Checklist 10, *WHO-5* WHO-5 Well-Being Index, *GSRS* Gastrointestinal Symptom Rating Scale, *HDL* High Density Lipoprotein, *LDL* Low Density Lipoprotein

**Table 2:** Daily intake of total energy, energy-yielding nutrients, non-nutritive sweeteners, vitamins and salt

Daily dietary intake	Median	Range
Energy		
Total energy (kJ)	9737	2648 - 21,816
Protein (g)	109	40 - 212
Fat (g)	90	21 - 283
Carbohydrates (g)	251	65 - 903
Sugar (g)	26	1 - 632
Non-nutritive sweeteners (NNS) (unit^a^)		
NNS total	3.3	0.0 – 43.0
NNS carbonated beverages	0.4	0.0 – 40.0
NNS non-carbonated beverages	0.1	0.0 – 32.0
NNS sweeteners in coffee and tea	0.0	0.0 – 27.0
Vitamins and salt		
Vitamin A (μg)	1341	352 - 4460
Vitamin B1 (mg)	2.6	0.8 – 7.8
Vitamin B2 (mg)	3.0	1.1 – 8.8
Vitamin B6 (mg)	2.7	0.9 – 10.0
Vitamin B12 (μg)	9.3	3.0 – 33.7
Vitamin C (mg)	170	11 - 623
Vitamin D (μg)	12.5	2.2 – 44.6
Folic acid (μg)	391	131 – 1077
β-carotene (μg)	4947	340 – 24,306
Salt (g)	7.5	2.4 – 18.8

^a^ *NNS* One unit = 100 ml beverages with NNS or 2 units of NNS for coffee/ tea

**Table 3:** Associations between non-nutritive sweeteners (dependent variable) and subjects’ characteristics

Patient characteristics	NNS total		NNS carb. beverages		NNS non-carb. beverages		NNS sweeteners	
	B	*p*-value	B	*p*-value	B	*p*-value	B	*p*-value
Gender (female/male)	−0.10	0.838	0.519	0.285	−0.049	0.924	−2.896	**0.005**
Age (years)	−0.04	0.073	−0.014	0.544	−0.045	0.063	0.002	0.951
BMI (kg/m2)	−0.025	0.640	−0.007	0.902	−0.032	0.582	0.040	0.510
Living with someone	−0.728	0.151	−0.030	0.953	−0.981	0.060	−0.011	0.985
Working	−0.124	0.594	0.383	0.114	−0.379	0.126	−0.047	0.858
Smoking	0.194	0.485	0.196	0.492	−0.043	0.884	−0.110	0.715
Perceived general health	0.011	0.965	0.070	0.793	0.098	0.722	0.083	0.771
Total physical activity	−0.184	**0.029**	−0.086	0.308	−0.030	0.732	0.014	0.883
Heavy physical activity	−0.477	**0.011**	−0.368	0.052	−0.212	0.278	0.116	0.576
Hypertension	0.201	0.607	0.261	0.518	−0.128	0.759	−0.340	0.442
Diabetes	0.971	**0.039**	0.639	0.174	1.227	**0.012**	0.171	0.748
Fibromyalgia	0.696	0.131	0.202	0.664	0.718	0.132	0.568	0.249
Muscle-skeletal pain score (range 0-12)	−0.004	0.952	0.001	0.987	−0.097	0.149	0.058	0.397
HSCL10 > 1.85	0.073	0.855	−0.194	0.639	−0.664	0.137	−0.028	0.951
WHO-5 (poor wellbeing)	0.452	0.249	0.805	**0.046**	−0.135	0.746	−0.297	0.509
Fatigue	1.232	**0.001**	0.490	0.184	0.316	0.408	0.575	0.159
IBS	0.317	0.444	−0.193	0.651	0.207	0.633	−0.047	0.915
Functional bloating	−0.379	0.486	0.600	0.280	−0.714	0.258	−1.365	0.067
Functional diarrhea	NA	NA	NA	NA	NA	NA	NA	NA
GSRS-diarrhea (score)	0.625	**0.009**	0.178	0.447	0.176	0.467	0.626	**0.012**
GSRS-bloating (score)	−0.112	0.509	−0.033	0.849	−0.324	0.084	−0.184	0.320
Blood tests								
Haemoglobin (g/dl)	−0.299	0.149	−0.063	0.765	−0.625	**0.007**	−0.534	**0.022**
Serum iron (μmol/L	−0.015	0.648	−0.042	0.237	−0.011	0.768	−0.049	0.205
Transferrin saturation	−0.875	0.673	−0.025	0.255	−0.011	0.631	−0.033	0.167
Ferritin (μg/dL)	0.002	0.311	−0.001	0.450	0.000	0.909	−0.001	0.615
CRP (mg/L)	0.040	0.226	0.029	0.393	0.081	**0.020**	0.042	0.228
s-Glucose (mmol/L)	0.110	0.082	0.122	0.055	0.044	0.492	0.005	0.944
HbA1C (%)	0.294	0.052	0.235	0.117	0.367	**0.018**	0.076	0.644
c-peptide (nmol/L)	0.662	**0.005**	0.410	0.077	0.279	0.244	0.509	0.052
Cholesterol (mmol/L)	0.116	0.542	0.188	0.339	−0.043	0.830	−0.096	0.661
HDL (mmol/L)	−0.612	0.291	−0.164	0.782	−1.017	0.115	0.541	0.394
LDL (mmol/L)	0.164	0.427	0.164	0.438	−0.026	0.907	−0.179	0.457
Vitamin A (μmol/L)	0.291	0.536	−0.725	0.136	−0.232	0.644	0.050	0.921
Vitamin B1 (nmol/L)	0.008	0.243	0.006	0.356	0.001	0.876	0.007	0.363
Vitamin B6 (nmol/L)	0.004	0.479	0.005	0.407	−0.011	0.178	−0.002	0.759
Vitamin B12 (pmol/L)	−0.001	0.384	0.000	0.727	0.000	0.940	−0.001	0.503
Vitamin D (nmol/L)	0.006	0.458	0.012	0.166	0.001	0.952	0.000	0.966
Folic acid (nmol/L)	−0.024	0.269	−0.007	0.759	−0.021	0.362	−0.018	0.433

*HSCL10* Hopkins Symptom Checklist 10, *WHO-5* WHO-5 Well-Being Index, *IBS* Irritable bowel syndrome, *GSRS* Gastrointestinal Symptom Rating Scale, *HDL* High Density Lipoprotein, *LDL* Low Density Lipoprotein

The analyses have been performed with ordinal logistic regression analyses adjusted for gender, age and BMI)

**Table 4:** Associations between the intake of NNS and intake of energy, energy-yielding nutrients, vitamins and salt

Diet	NNS Total		NNS Carbonated		NNS Non-carb		NNS Sweeteners	
	rho	*p*-value	rho	*p*-value	rho	*p*-value	rho	*p*-value
Total energy (kcal)	0.138	**0.003**	0.235	**0.004**	- 0.101	**0.0329**	0.014	0.080
Protein (g)	0.081	0.106	0.198	**0.012**	- 0.066	0.551	- 0.007	**0.028**
Fat (g)	0.172	**0.013**	0.273	**0.005**	- 0.053	**0.043**	0.083	0.094
Carbohydrates (g)	0.145	**0.002**	0.221	**0.014**	- 0.097	**0.031**	- 0.048	**0.031**
Sugar (g)	0.204	**0.003**	0.257	**0.003**	- 0.037	0.091	- 0.111	**0.012**
Vitamin A (μg)	- 0.242	**0.001**	- 0.092	0.077	- 0.185	**0.014**	- 0.016	0.659
Vitamin B1 (mg)	- 0.076	0.062	- 0.017	0.121	- 0.171	0.088	0.025	0.595
Vitamin B2 (mg)	- 0.092	0.060	- 0.016	0.088	- 0.190	0.053	0.026	0.054
Vitamin B6 (mg)	- 0.033	0.238	- 0.005	0.111	- 0.091	0.558	0.060	0.611
Vitamin B12 (μg)	0.027	0.804	0.103	0.584	- 0.017	0.622	0.066	0.595
Folic acid (mg)	- 0.028	0.074	0.043	**0.036**	- 0.150	0.160	0.065	0.730
β-Carotene (μg)	- 0.154	0.091	- 0.084	0.145	- 0.175	**0.033**	0.107	0.428
Vitamin C (mg)	- 0.194	**0.002**	- 0.050	0.051	- 0.172	**0.026**	0.084	0.083
Vitamin D (μg)	- 0.198	**0.016**	- 0.146	**0.033**	- 0.217	0.079	- 0.052	0.069
Salt (g)	0.261	**0.001**	0.321	**<0.001**	0.051	**0.001**	0.070	**0.028**

*NNS* Non-Nutritive Sweeteneres

The correlations have been calculated with Spearmans’ rho, and the p-values with ordinal logistic fractional polynomial regression adjusted for gender, age and BMI

## Discussion

The study confirms the findings from studies in the general population that the use of NNS is high in overweight and obese adults [18–21]. Half of the subjects used more than 3.3 units of NNS per day, which corresponds to 330 ml beverages with NNS. An intake of 2 – 4 l was not uncommon.

The main finding was the associations between NNS and an unhealthy lifestyle. In literature, less is known about these clinically relevant outcomes than about the weight. In this study, NNS was associated with a less healthy diet, reduced physical activity, low well-being and fatigue, which indicate an unhealthy lifestyle. The results indicate that the intake of NNS-containing beverages was approximately 100 ml higher in subjects with diabetes than in those without, and the same difference was seen between those with strong physical activity less than 1 hour/week compared to those with more than 2 h, and in subjects with low mood. The clinical significance of these effects are uncertain, but is indicative of an unhealthy lifestyle associated with the use of NNS.

A high intake of NNS was associated with increased intake of fat, proteins, carbohydrates including sugar, and salt; and reduced intake of some vitamins. The high intake of energy is harmful to obese subjects. The association with the intake of sugar could support the hypothesis that NNS encourage sugar craving and dependence by an altered metabolism and processing of sweet taste in the brain [22, 23]. Most of the unfavorable associations were related to the use of NNS in carbonated beverages, probably because the highest intake of NNS was from carbonated beverages. The stongest correlations were between intake of NNS containing beverages and salt. It is likely that these users combine the beverages with intake of salted food and snacks, which has also been shown by others [18]. Most of the associations between intake of NNS and energy and nutrients were weak (rho <0.2) and NNS explain only a minor part of the variation. The negative associations between intake of NNS and c-peptid, HbA1c and perhaps also Hb might have been confounded by diabetes. To adjust the analyses for all comorbidity including diabetes, in addition to age, gender and BMI was judged as inappropriate. The users of NNS in non-carbonated beverages, tea, and coffee seem to have a more conscious and correct use of NNS with a slightly reduced intake of total energy, carbohydrates, and sugar. They also reduced the intake of β-Carotene and vitamin C, indicating that they reduced all kinds of food including the healthy fruits and vegetables. Opposed to the findings in this study, population-based studies in the UK, US and Canada suggest a higher dietary quality in NNS consumers than in nonconsumers [19, 20]. The way NNS are used and the physiological and psychological effect of NNS might differ between subjects randomly selected from the population and subjects referred for treatment of morbid obesity at a spesialised hospital unit. Although NNS have been accused of a diabetogenic effect, the associations between NNS and diabetes and c-peptide in this study are probably explained by the higher use of NNS by subjects with diabetes [24, 25].

Reduced physical and mental health was also associated with NNS. The users of NNS had a feeling of poor well-being and more fatigue, and were less physically active. These aspects have not been focused on in literature as far as we know. Caffeine- and NNS-containing beverages might have been used to counteract fatigue and as an excuse for less physical activity. Diarrhea associated with NNS for use in coffee and tea might have been an adverse event related to some of the NNS.

The association between the use of NNS and BMI is not clear [8, 26]. The lack of associations between the use of NNS and BMI in this study was likely because all subjects were morbidly obese, but could indicate a lack of weight-reducing effect of NNS. In population-based observational studies, the use of NNS is higher in overweight and obese subjects than in healthy-weight subjects [18–20]. The findings could indicate that NNS induce weight gain, but it more likely reflects the use of NNS for weight reduction by overweight and obese subjects.

Numerous studies from agriculture, in the laboratory and in humans indicate a counterintuitive effect of NNS with increased food intake and body weight, accumulation of fat, weaker caloric compensation, metabolic syndrome and cardiovascular diseases [27–29]. Animal studies have shown weight gain and metabolic dysregulation after intake of NNS [29, 30]. NNS are not inert substances, and physiological effects on metabolism and energy balance have been proposed to explain an unexpected weight-inducing effect in long-term follow-up studies in children and adults [7, 31–33]. NNS affect the glucose metabolism and have been associated with type 2 diabetes [24, 34–36]. Concerns have also been raised about effects on appetite, eating behaviour, satiation, satiety, craving, reward, addiction, cognitive functions, neurophysiology, and brain function [22, 23, 37–40].

More recently, the effect of NNS on the gut microbiome has achieved considerable attention. The disturbed gut-brain interaction caused by the NNS-induced dysbiosis might in part explain the effects associated with obesity such as weight gain, metabolic changes including glucose intolerance, neurophysiological and psychological changes [41–43].

Except for a slightly favourable effect in the subgroup of subjects using NNS-containing non-carbonated beverages, the overall findings were discouraging. It was anticipated that subjects who were referred for obesity and therefore motivated for weight-reducing interventions, had a conscious relation to the use of NNS as a way to reduce energy intake. Most of them had bariatric surgery later on.

Despite numerous concerns and an extensive literature, the correct use of NNS is unknown [25]. The actual knowledge has been summarized by the U.S. Department of Health and Human Services and U.S. Department of Agriculture in “Dietary Guidelines for Americans 2015-2020”: “*…. replacing added sugar with high-intensity sweeteners may reduce calorie intake in the short-term, yet questions remain about their effectiveness as a long-term weight management strategy*”, and “*Based on available scientific evidence, these high-intensity sweeteners have been determined to be safe for the general population*” [44]. Shankar et al. gave an intelligent advice *“…for optimal health it is recommended that only minimal amounts of both sugar and NNS be consumed”* [45].

### Strengths and limitations

The focus on an unselected group of consecutive subjects with morbid obesity from a general hospital and their health and lifestyle, and not on overweight and obesity in general and body weight only, was a strength. This study from a general hospital is likely to be representative of unselected consecutive subjects referred to a specialized unit for morbid obesity. The validity of the results for all subjects with overweight and obesity is unknown. The lack of information about the use of NNS in other products than beverages and the different types of NNS was a limitation. The FFQ only asked for the use of NNS-containing carbonated beverages, non-carbonated beverages and units of NNS in tea and coffee and not the specific products. Information about NNS in packets added to other beverages or food was not asked for. The limited sample size reduces the ability to control for confounders. No correction was performed for the numerous correlations, which increased the risk of type I errors.

## Conclusions

The use of NNS-containing beverages in subjects with morbid obesity was associated with an unhealthy lifestyle, reduced physical and mental health, and unfavourable dietary habits. Lifestyle and dietary advice are therefore particularly important to subjects with morbid obesity using NNS-containing beverages. There were no significant associations between the use of NNS-containing beverages and BMI. The study gave no support for the recommendation of NNS-containing beverages to subjects with morbid obesity.

## Abbreviations

BMI: Body Mass Index
CI: Confidence interval
FFQ: Food Frequency Questionnaire
GSRS: Gastrointestinal Symptom Rating Scale
HDL: High-Density Lipoprotein
HSCL-10: Hopkins Symptoms Checklist 10
IBS: Irritable Bowel Syndrome
LDL: Low-Density Lipoprotein
NNS: Non-Nutritive Sweeteners
SD: Standard Deviation
WHO: World Health Organization
